# A high-throughput screen identifying sequence and promiscuity characteristics of the *loxP  *spacer region in Cre-mediated recombination

**DOI:** 10.1186/1471-2164-7-73

**Published:** 2006-04-04

**Authors:** Perseus I Missirlis, Duane E Smailus, Robert A Holt

**Affiliations:** 1 Genome Sciences Centre, BC Cancer Agency, Suite 100, 570 West 7th Ave, Vancouver, BC, V5Z 4S6, Canada

## Abstract

**Background:**

Cre-*loxP *recombination refers to the process of site-specific recombination mediated by two *loxP *sequences and the Cre recombinase protein. Transgenic experiments exploit integrative recombination, where a donor plasmid carrying a *loxP *site and DNA of interest integrate into a recipient *loxP *site in a target genome. Unfortunately, integrative recombination is highly inefficient because the insert is flanked by two *loxP *sites, which themselves become targets for Cre and lead to subsequent excision of the insert. A small number of mutations have been discovered in parts of the *loxP *sequence, specifically the spacer and inverted repeat segments, that increase the efficiency of integrative recombination. In this study we introduce a high-throughput *in vitro *assay to rapidly detect novel *loxP *spacer mutants and describe the sequence characteristics of successful recombinants.

**Results:**

We created synthetic *loxP *oligonucleotides that contained a combination of inverted repeat mutations (the *lox*66 and *lox*71 mutations) and mutant spacer sequences, degenerate at 6 of the 8 positions. After *in vitro *Cre recombination, 3,124 recombinant clones were identified by sequencing. Included in this set were 31 unique, novel, self-recombining sequences. Using network visualization tools, we recognized 12 spacer sets with restricted promiscuity. We observed that increased guanine content at all spacer positions save for position 8 resulted in increased recombination. Interestingly, recombination between identical spacers was not preferred over non-identical spacers. We also identified a set of 16 pairs of *loxP *spacers that reacted at least twice with another spacer, but not themselves. Further, neither the wild-type P1 phage *loxP *sequence nor any of the known *loxP *spacer mutants appeared to be kinetically favoured by Cre recombinase.

**Conclusion:**

This study approached *loxP *spacer mutant screening in an unbiased manner, assuming nothing about candidate *loxP *sites save for the conserved 4 and 5 spacer positions. Candidate sites were free to recombine with any other sequence in the pool of all possible sites. The subset of *loxP *sites identified here are candidates for *in vivo *serial recombination as they have already demonstrated limited promiscuity with other *loxP *spacer and stability in the presence of Cre.

## Background

Cre-*loxP *recombination is an important tool in molecular genetics. Cre ("Causes recombination") recombinase from bacteriophage P1 recognizes a specific 34 bp target sequence, termed *loxP*, composed of an 8 bp spacer region flanked by two identical 13 bp inverted repeats [1] (Table 1). Each base in the spacer region is conventionally named 1,2,3,4,5,6,7,8 according to its order in the sequence. Cre-*loxP *sites mediate site specific intra- or inter-strand exchange of DNA molecules catalyzed by Cre recombinase. Depending on the location and the orientation of these sites, they can invert, insert, delete or exchange fragments of DNA in prokaryotic or eukaryotic systems [2-5]. Orientation of insert DNA post-recombination is dependent on the orientation of the sites prior to the reaction, with sites in the same orientation on a given DNA strand mediating excision of intervening sequence and sites in opposite orientation mediating inversion of intervening sequence. Since the excision reaction is kinetically favoured over the insertion reaction, gene deletion/inactivation experiments are straightforward to engineer by flanking the target sequence with *loxP *sites. The difficulty in accomplishing DNA insertion is that the insertion reaction results in the presence of two *loxP *sites in *cis *post-recombination, which themselves become substrates for Cre and lead to rapid excision of the inserted segment.

**Table 1:** Published mutant *loxP *sites

***loxP *site**	**Left inverted repeat sequence**	**Spacer (5'→3')**	**Right inverted repeat sequence**
**Wild-type**	ATAACTTCGTATA	ATGTATGC	TATACGAAGTTAT
***lox *511 [6]**	ATAACTTCGTATA	ATGTATaC	TATACGAAGTTAT
***lox *5171 [7]**	ATAACTTCGTATA	ATGTgTaC	TATACGAAGTTAT
***lox *2272 [7]**	ATAACTTCGTATA	AaGTATcC	TATACGAAGTTAT
***m2 *[8]**	ATAACTTCGTATA	AgaaAcca	TATACGAAGTTAT
***m3 *[8]**	ATAACTTCGTATA	taaTAcca	TATACGAAGTTAT
***m7 *[8]**	ATAACTTCGTATA	AgaTAgaa	TATACGAAGTTAT
***m11 *[8]**	ATAACTTCGTATA	cgaTAcca	TATACGAAGTTAT
***lox *71 [12, 13]**	taccgTTCGTATA	ATGTATGC	TATACGAAGTTAT
***lox *66 [12, 13]**	ATAACTTCGTATA	ATGTATGC	TATACGAAcggta

Two classes of variant *loxP *sites are available to engineer stable Cre-*loxP *integrative recombination. Both exploit sequence mutations in the Cre recognition sequence, either within the 8 bp spacer region or the 13-bp inverted repeats. Spacer mutants such as *lox*511[6], *lox*5171 [7], *lox*2272 [7], *m2, m3, m7*, and *m11 *[8] (Table 1) recombine readily with themselves but have a markedly reduced rate of recombination with the wild-type site. This class of mutants has been exploited for DNA insertion by Recombinase Mediated Cassette Exchange (RMCE) [9-11]. Inverted repeat mutants represent the second class available and contain altered bases in the left inverted repeat (LE mutant) or the right inverted repeat (RE mutant). The LE mutant, *lox*71, has 5 bp on the 5' end of the left inverted repeat that are changed from the wild type sequence to TACCG [12,13] (Table 1). Similarly, the RE mutant, *lox*66, has the five 3'-most bases changed to CGGTA (Table 1). Inverted repeat mutants are used for integrating plasmid inserts into chromosomal DNA with the LE mutant designated as the "target" chromosomal *loxP *site into which the "donor" RE mutant recombines. Post-recombination, *loxP *sites are located in *cis*, flanking the inserted segment. The mechanism of recombination is such that post-recombination one *loxP *site is a double mutant (containing both the LE and RE inverted repeat mutations) and the other is wild type [14-16]. The double mutant is sufficiently different from the wild-type site that it is unrecognized by Cre recombinase and the inserted segment is not excised. Recently, spacer and inverted repeat mutants have been combined to increase the specificity and stability of integrative recombination [8,17].

In past experiments, novel spacer mutants were discovered by mutating a suite of bases [6,7] or generating a set of potential spacer mutants and testing recombination between these spacers with the wild-type *loxP *site [8]. Lee and Saito used an *in vitro *assay that evaluated the recombination efficiency of 24 spacers with 1 bp substitutions and 30 spacers with 2 bp substitutions from the sequence of the wild-type *loxP *[7]. Their data suggested that homology was required at positions 2–5 and positions 6–7 for efficient strand exchange and resolution of the Holiday junction whereas positions 1 and 8 had relaxed homology requirements. They concluded that homology was essential to achieve recombination rates between mutant *loxP *spacers comparable to that of the wild-type sequence. Their success with the *lox*2272 mutant suggested that positions 2 and 7 were particularly important in blocking promiscuous recombination. Langer *et al. *focused on the *in vivo *functionality of novel *loxP *sites in bacterial and eukaryotic systems [8]. In their work, a suite of random *loxP *sites were generated by utilizing a template *loxP *oligonucleotide with degenerate spacers at positions 1,2,3,6,7,8. They discovered four spacers with vigorous homologous recombination and limited recombination with the wild-type sequence (*m2, m3, m7, m11*). Only the *m2 *mutant was tested and found to be functional for RMCE in bacteria and Cre-expressing eukaryotic cells (HEK-293). Other studies have evaluated the *in vivo *heterospecificity of the wild-type spacer versus other published *loxP *sequences. Siegel *et al. *tested heterospecific recombination between five published *loxP *spacer mutants and found that wild-type/FAS, wild-type/2272 and 2272/5171 pairings were incompatible while limited compatibility was found between wild-type/511 sites [18]. However, another study found equal recombination efficiency into a BAC between the wild-type and *lox*511 spacers [19]. Recently, a phage P1 transduction assay recapitulated the sensitivity and exclusivity of the *lox*2272 and *lox*5171 spacer mutants [20]. However, to the best of our knowledge, no studies have investigated if the wild-type spacer is the most efficient sequence for Cre-mediated recombination.

Given that spacer and inverted repeat mutants have been used together successfully, it may be possible to introduce numerous DNA segments into a given target molecule, chromosome or genome if a sufficient number of non-promiscuous LE/RE-spacer mutants can be identified. In the present study we have developed and executed a high-throughput *in vitro *strategy to rapidly identify novel LE/RE-spacer mutants and determine their specificity.

## Results

### The recombination reaction

**Figure 1:**
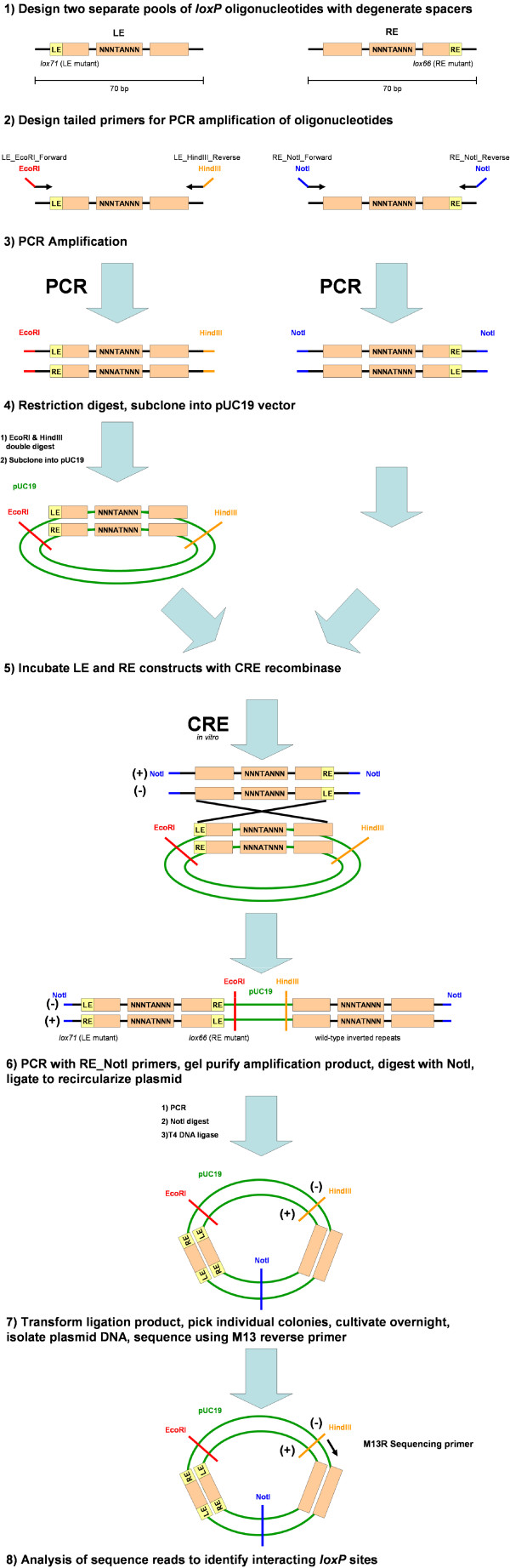
**Experimental steps for high-throughput LE/RE-spacer mutant *loxP *screen**. Two pools of oligonucleotides containing LE and RE mutants were PCR-amplified to create dsDNA. The LE-mutants were subcloned in pUC19. LE-mutants (in plasmid form) were combined with linear RE oligonucleotides in the presence of Cre recombinase overnight to form the expected 1.8 kb recombination products. These products were PCR amplified with the NotI tailed forward and reverse primers, gel-purified, digested with NotI, re-circularized to reform the pUC19 plasmid and then sequenced.

Two oligonucleotides (LE, RE) were designed that contained *loxP *sites with six degenerate spacer nucleotides (positions 1,2,3,6,7,8) and two central fixed spacer nucleotides (4 and 5) (Figure 1, Table 2). The central nucleotides in the 4, 5 position were limited to thymine and adenine residues because these have been previously suggested by mutational analysis to be essential for strand exchange [6]. One of the oligonucleotide pools (LE) contained the *lox*71 left arm mutant sequence and the other (RE) contained the *lox*66 right arm mutant sequence (Figure 1, Table 2) [12]. These oligonucleotides were converted to double stranded product by PCR. Primers used to generate the LE PCR product were tailed with EcoRI and HindIII restriction sites for subcloning into the pUC19 vector and primers used to generate the RE PCR product were tailed with NotI restriction sites (Table 2). These two pools of oligonucleotides were then incubated in the presence of Cre recombinase (Figure 1).

**Table 2:** Oligonucleotide sequences (LE, RE) and primers used in the high-throughput LE/RE-spacer mutant *loxP *screen

**Name**	**Sequence**
**LE**	5' < CTGAGCTCGGGTGCCTGTTACCGTTCGTATANNNTANNNTATACGAAGTTATGACTGCACCCATCAGAGC > 3'
**LE_EcoRI_Forward**	5' < TGACGAATTCCTGAGCTCGGGTGCCTGT > 3'
**LE_HindIII_Reverse**	5' < GCTAAAGCTTGCTCTGATGGGTGCAGTC > 3'
**RE**	5' < TGTCTCTGGCACGCTAGGATAACTTCGTATANNNTANNNTATACGAACGGTATATGGCCTTCTGTAGCGG > 3'
**RE_NotI_Forward**	5' < GTACGCGGCCGCTGTCTCTGGCACGCTAGG > 3'
**RE_NotI_Reverse**	5' < GTACGCGGCCGCCCGCTACAGAAGGCCATA > 3'

### Sequencing and analyzing the successful recombinants

The desired 1.8 kbp recombination products were amplified by PCR using the RE_NotI forward and reverse primers (Table 2) then digested with NotI, agarose gel purified and re-circularized with T4 DNA ligase to generate a library of paired *loxP *recombination products in pUC19 vector (Figure 1). These plasmids were transformed into DH10B cells, grown overnight, and plated on solid media. Each individual colony (clone) represented a distinct, successful, recombination reaction between two *loxP *spacer sequences. A total of 5,670 clones were picked, grown overnight, and plasmid DNA was isolated and sequenced with M13 Reverse sequencing primer using previously described methods (Figure 1) [21]. Of these clones, 4,992 yielded successful sequence (See Additional files 1 &2).

**Figure 2:**
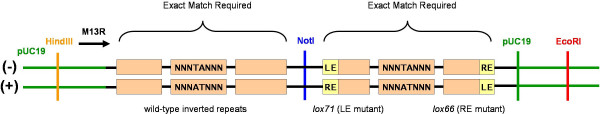
**Sequence features of a typical read**. Sequence was generated from the negative strand with the M13 Reverse primer (M13R). Exact matches to the wild-type and *lox*71 and *lox*66 inverted repeat mutants were required for spacers to proceed to further analysis. The NotI site, pUC19 vector boundary and EcoRI were also detectable. The HindIII site could not be detected with the M13 Reverse primer.

The M13 Reverse sequencing primer generated data from the negative strand (Figure 1, Figure 2). We sequenced off of the negative strand because the position of the M13 Forward primer was too close to the *lox*71/*lox*66 double mutant to reliably generate the sequence of both spacers. A limited number of clones (768) were sequenced in the positive orientation but only 42/768 produced high quality spacer sequences (See Additional files 1 &2) all of which agreed with that of the negative strand.

**Figure 3:**
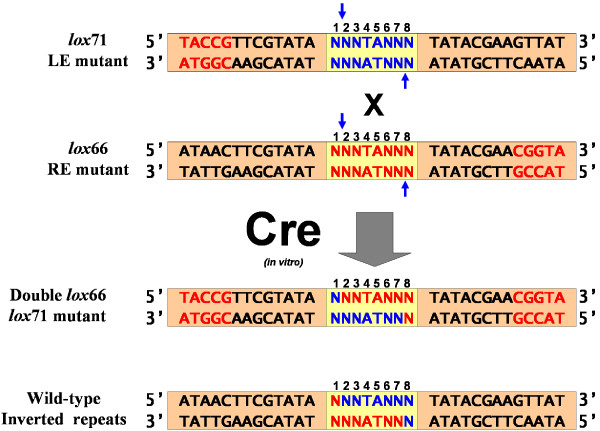
**Mechanism of recombination between mutant *loxP *spacers**. Input *lox*71 left inverted repeat mutant (LE) and *lox*66 right inverted repeat mutant (RE) oligonucleotide sequences with scissile phosphodiester bonds highlighted with blue arrows. Cre-mediated recombination between the LE mutant and RE mutant resulted in one *loxP *site containing both mutants and another with wild-type inverted repeats. The input LE/RE spacer mutants of the reacting oligonucleotides were inferred by working backwards from the location of the scissile bonds in the final sequenced recombinant.

According to the reaction mechanism, we expected to see wild-type inverted repeats flanking one spacer and the *lox*66 and *lox*71 inverted repeats flanking the other spacer post-recombination (Figure 3) [12,13]. Consequently, a typical sequencing read was composed of the following sequence features (median feature location from read start given in bp): left wild-type inverted repeat (14 bp), first spacer (27 bp), right wild-type inverted repeat (34 bp), NotI site (65 bp), *lox*71 inverted repeat mutant (91 bp), second 8 bp spacer (104 bp), lox *66 *inverted repeat mutant (111 bp), start of the pUC19 vector (133 bp) and EcoRI site (143 bp) (Figure 2). Successful recombination reactions were defined as those sequences that contained exact matches to the wild-type inverted repeat sequences flanking an 8 bp spacer (ATTACTTCGTATA NNNNNNNN TATACGAAGTTAT) and the *lox*66, *lox*71 inverted repeat mutations flanking an 8 bp spacer (TACCGTTCGTATA NNNNNNNN TATACGAACGGTA) (Figure 2). Five spacers lacked the central TA nucleotides but were retained in the analysis because they successfully recombined.

There were 3,124 reverse strand sequence reads from successful recombination reactions that were used for further analysis. However, these sequences could not be analyzed as is. First, each spacer was reverse complemented to facilitate comparisons with published *loxP *spacers as most spacers are published in the positive strand orientation [6,7]. Since each sequence read represented the final product of recombination, the spacer sequence of the original input LE and RE oligonucleotides had to be inferred based on the published location of the scissile bonds [22] and mechanism of recombination (Figure 3) [14-16]. For each recombination reaction, the input LE oligonucleotide spacer was defined as *loxP *spacer position 1 from the LE/RE double inverted repeat mutant and positions 2–8 from the spacer with wild-type inverted repeats (Figure 3). Accordingly, the input RE oligonucleotide spacer was defined as position 1 from the wild-type inverted repeat *loxP *spacer sequence and positions 2–8 of the LE/RE double inverted repeat mutant (Figure 3).

**Figure 4:**
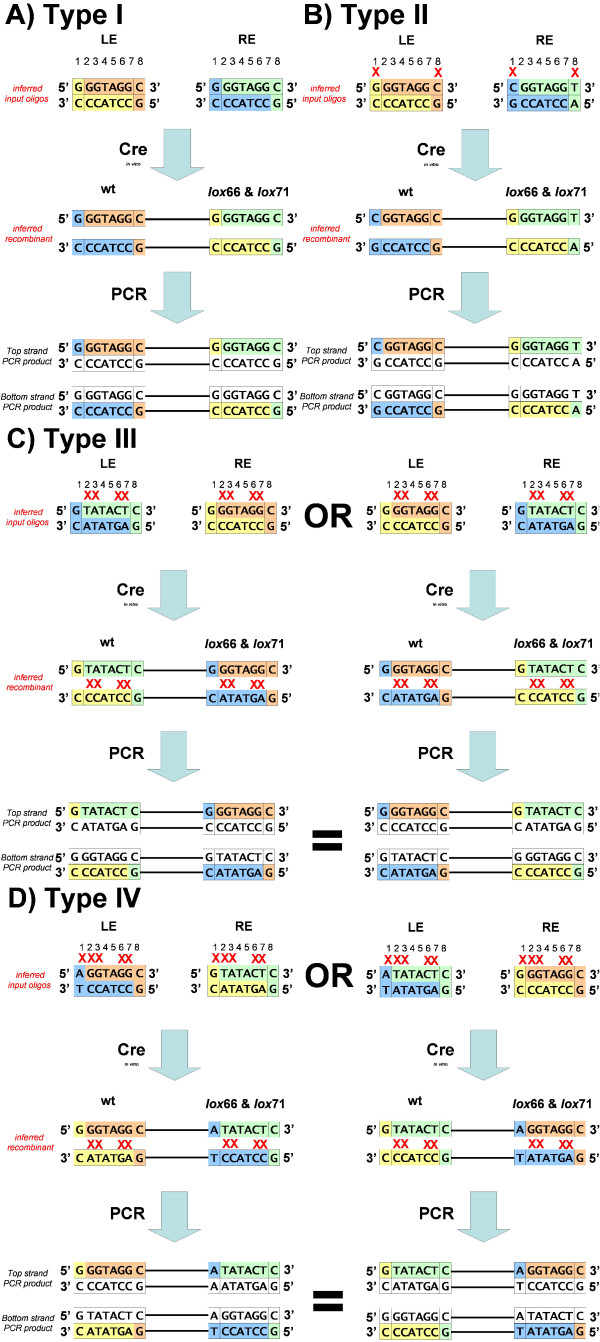
**Schematics of Type I-IV mismatch reactions**. Diagrammatic representation of how the four categories of input oligonucleotides were inferred. Recombination segments originating from the LE and RE oligonucleotides are highlighted with the same colour. Each initial recombinant molecule, when PCR amplified, produced two pools of double-stranded sequence; one from amplification of the positive strand and the other from amplification of the negative strand (Figure 1, steps 5 & 6). Since we sequenced and analyzed these PCR products in the 5'→3' orientation, rather than the actual recombinant molecule, we ensured that unambiguous input oligonucleotides were inferred for further analysis. A) Type I and B) Type II. These classes are defined as having either identical input spacers (Type I) or input spacers mismatched at positions 1 and/or 8 (Type II). In both types the original oligonucleotides could be inferred definitely. C) Type III. This class contains input spacers with different bases at positions 2,3,6 and 7. In all spacers positions 4,5 are fixed as TA during oligonucleotide synthesis. In these cases the spacer sequences could be identified but their origin within the LE or RE oligonucleotides was ambiguous. Regardless of initial oligonucleotide orientation, these mismatches resulted in two distinct recombinant molecules that gave rise to the same two pools of sequence reads. D) Type IV. This class contains input oligonucleotides with different bases at position 1 and/or 8 plus a mismatch at one or more of positions 2,3,6 and 7. In these cases the input spacer sequences are ambiguous as two possible recombinants arising from different sets of LE, RE oligonucleotides can give rise to the same pool of sequence reads. Mismatches between reacting spacers are indicated by red "X" marks in the input oligonucleotides

However, there remained a further layer of complexity in the data. Each recombinant DNA molecule derived from mismatching spacers gave rise to two pools of PCR products from the same PCR reaction (Figure 1, steps 5 & 6), one pool derived from amplification of the positive strand of the initial recombinant molecule and another from the negative strand. From each of these two types of PCR products we sequenced the top, or positive strand. Since we could only observe the sequence of these final PCR products, we had to infer the sequence of input oligonucleotides using the established *loxP *recombination mechanism [14-16]. We defined four categories of inferred input oligonucleotides based on the location of the mismatched base(s) (Table 3) (Figure 4). The Type I class (Figure 4A) corresponded to identical spacers in the two input oligonucleotides that yielded a recombinant molecule with identical spacers. For this class, the PCR products and sequence reads originating from the positive versus negative strand of the original recombinant molecule were identical. The Type II class (Figure 4B) corresponded to input oligonucleotides with discrepancies in positions 1 and/or 8 in their reacting spacers. For this class the input oligonucleotides can be unambiguously assigned as well because positions 1 and 8 are not exchanged between strands during recombination. The Type III class (Figure 4C) corresponded to input oligonucleotides with one or more mismatches in positions 2 through 7, which are the bases that undergo strand exchange during recombination. For Type III, the sequence of the input spacers could be inferred but the origin within the LE or RE oligonucleotides was ambiguous. Lastly, the Type IV class (Figure 4D) corresponded to input oligonucleotides that had mismatches in positions 1 or 8, and also had one or more mismatches in positions 2 through 7. For this class the precise identity of the input oligonucleotides was ambiguous because two different pairs of oligonucleotides could produce the same PCR products and sequence reads. Due to this ambiguity, this category was excluded from the majority of analyses.

**Table 3:** Total reactions and mutations per position in the 8 bp *loxP *spacer region organized by mismatch Type

**Type**	**Pos 1**	**Pos 2**	**Pos 3**	**Pos 4**	**Pos 5**	**Pos 6**	**Pos 7**	**Pos 8**	**Total Reactions**
**Type I**	0	0	0	0	0	0	0	0	47
**Type II**	209	0	0	0	0	0	0	244	303
**Type III**	0	144	126	1	0	167	143	0	284
**Type IV**	1,575	1,361	1,104	3	1	1,349	1,331	2,128	2,490
**Total**	**1,784**	**1,505**	**1,230**	**4**	**1**	**1,516**	**1,474**	**2,372**	**3,124**

A Perl script (see Additional file 3) was run on these sequences to transform them to their inferred original format and all intermediates based on the reaction mechanisms (Figure 3) (see Additional file 4). There were a total of 47 Type I reactions, 303 Type II reactions, 284 Type III reactions and 2,490 Type IV reactions with an average number of mismatches/spacer of 0, 1.5, 2.05 and 3.56 respectively (Table 3). The minimum number of mismatches varied by type: zero in Type I or perfect matches, 1–2 in Type II, 1–4 in Type III, and 2–6 in the Type IV. Type II reactions had an approximately equal number of single and double mismatches (150 and 153 respectively). Type III reactions had 94 single mismatches, 102 double mismatches, 60 triple and 19 quadruple mismatches. Lastly, Type IV reactions had 379, 878, 785, 368, and 80 of two, three, four, five and six bp mismatches, respectively. The observation of 80 Type IV reactions with six mismatches reflects the ability of the *loxP *spacer to tolerate numerous mutations and continue to recombine.

### Sequence characteristics of the Cre-loxP spacer region

**Figure 5:**
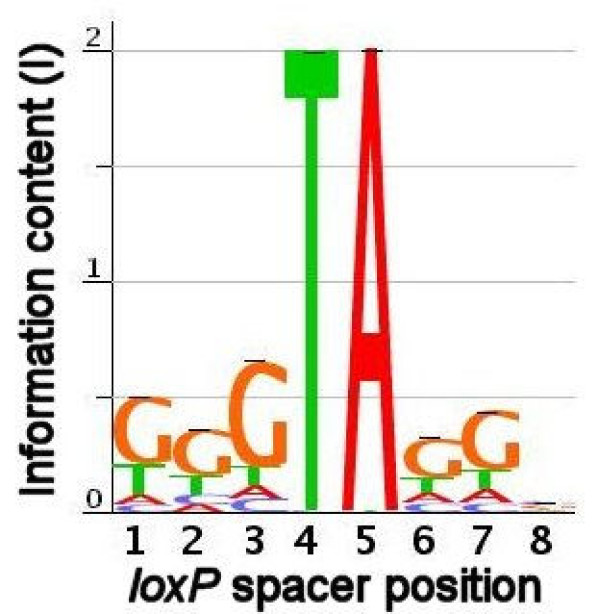
**Sequence logo representing the Cre-*loxP *consensus site**. A total of 6,248 spacers that participated in successful recombination reactions were used to generate a sequence "logo" representing the nucleotide preference of Cre recombinase in the spacer region. Information content (I) as each spacer position was calculated (see Methods for details). Base height (H) represents nucleotide frequency and at each position in the *loxP *spacer region.

**Figure 6:**
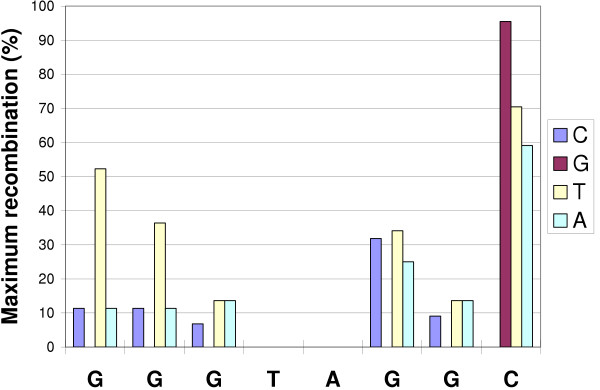
**Effect of nucleotide location and sequence within the spacer on recombination frequency**. Effect on recombination rate of changing one base at each degenerate spacer position from the sequence of the most promiscuous spacer (i.e. changing the base at position 1 from G to C creates the spacer CGGTAGGC which causes a 88% drop in total recombination relative to that of the most promiscuous spacer GGGTAGGC).

Cre-*loxP *recombination is mediated by the cooperative binding to the inverted repeat of two Cre proteins to each *loxP *site [1,14]. We used the suite of successful input spacer sequences to build a binding profile, in the same manner that sequences from a SELEX scan of a transcription factor are used to determine the consensus binding site [23]. We decided to use all types of spacers in our data for this analysis including Type IV. It is reasonable to include Type IV reactions in this analysis because the nucleotide composition at each position can be unambiguously determined and is all that is necessary for this analysis (Figure 4D). The 6,248 spacers (from 2 × 3,124 successful input LE and RE oligonucleotides) that successfully recombined showed no base preference at position 8, but a trend towards guanine at each of the remaining five degenerate positions (Table 4). A sequence logo [24] representing the consensus Cre-*loxP *spacer sequence is shown in Figure 5. Of all sites, the one most active in recombination was GGGTAGGC, which recombined with itself 6 times and also reacted with 26 other distinct spacer sequences in a total of 44 reactions. Further, we evaluated the effect that deviation from the most promiscuous spacer by 1 bp at each position had on recombination rate (Figure 6). As supported by the binding profile (Figure 5), position 8 was most impervious to substitutions with transversions from cytosine to guanine having the least effect. Transition to thymine and transversion to adenine had roughly the same effect dropping the recombination rate to around 65% of the maximum. Recombination rates dropped precipitously upon transitions of G to A or transversions to A at all positions with a slightly less pronounced effect at position 6. On the other hand, transversions to thymine were the most tolerated at each position save for position 3 where it mirrored the effect of C and A substitutions. Positions 3 and 7 appear to be the most sensitive overall to changes from the most promiscuous sequence.

**Table 4:** Base frequency (%) by position of each input *loxP *spacer sequence that successfully recombined

**Pos**	**1**	**2**	**3**	**4**	**5**	**6**	**7**	**8**	**Total**
**A:**	8.07%	9.31%	8.98%	0.00%	99.98%	12.36%	11.89%	17.13%	10,479
**T:**	25.88%	23.40%	11.72%	99.94%	0.02%	20.95%	18.61%	22.25%	13,918
**G:**	59.60%	55.67%	70.50%	0.00%	0.00%	55.27%	60.68%	28.30%	20,619
**C:**	6.45%	11.62%	8.80%	0.06%	0.00%	11.43%	8.82%	32.33%	4,968
**Total**	6,248	6,248	6,248	6,248	6,248	6,248	6,248	6,248	49,984

### Novel recombining LE/RE-spacer mutants and their promiscuity profile

**Figure 7:**
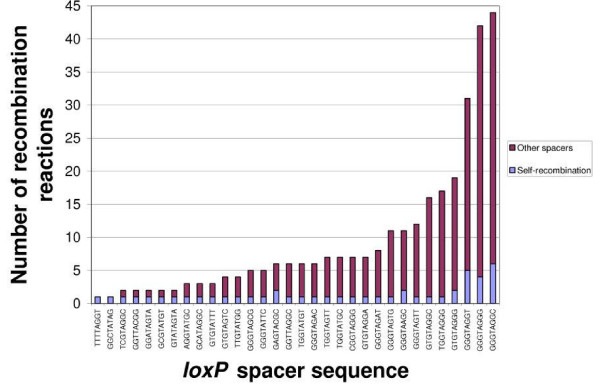
**Recombination profile of self-recombining *loxP *sites**. For each self-recombining spacer, the light blue portion of the stacked bar represents the count of self-recombination reactions and maroon the count of recombination reactions with other spacers.

Each sequence read had two, usually non-identical, *loxP *spacers representing a distinct recombination reaction. Thus, each spacer in the library had a promiscuity profile defined by the suite of *loxP *sites with which it recombined. As previously discussed, since Type IV mismatches had ambiguous input oligonucleotides they were not included in this section of the analysis (Figure 4D). Inferred spacer sequences were divided into two sets: self (a spacer sequence that recombined with itself plus one or more other spacer sequences) and non-self (a spacer sequence that did not recombine with itself, but did recombine with another non-self spacer more than once). For non-self recombining pairs, we screened for the subset that were replicated because the majority of spacer pairs were singleton non-self spacer pairs. The self and non-self sets are mutually exclusive. In the set of 3,124 successful recombination reactions, 32 self-recombining spacers were discovered (Figure 7). Of these, only one spacer AGGTATGC or *lox*23 has been described previously [7], the remaining 31 are novel self-recombining spacers. Spacers TTTTAGGT and GGCTATAG recombined solely with themselves but this exclusivity may be a reflection of limited sampling rather than a property of the spacer. Self-recombination events were correlated with increasing total recombination reactions (r^2 ^= 0.89, *p *< 0.05). The most promiscuous self-recombining sequence was GGGTAGGC which underwent 44 insertional recombination reactions. There were also a set of 16 non self-recombining spacers (Table 5). Of these TGGTAGGC and TGGTAGGT recombined with each other most frequently (4 reactions). The most promiscuous non self-recombining spacer was GGGTAGGA with 26 total reactions. We tested with the χ^2 ^statistic if each spacer from both the self and non-self sets recombined with equal frequency with all of their partners. All spacers did not significantly deviate from the null hypothesis of equal recombination frequency with all their partners save for GGGTAGGG (χ^2^, *p *< 0.05). The spacer GGGTAGGG deviated from the null hypothesis because it had higher promiscuity within a number of non-identical spacers rather than with itself (i.e. TGGTAGGC, GGGTAGGT, TGGTAGGA all recombined more frequently with GGGTAGGG than GGGTAGGG did with itself).

**Table 5:** Non-self recombining spacers

**LE**	**RE**	**Reactions**
TGGTAGGC	TGGTAGGT	4
GGGTATGC	GGGTATGG	3
GTGTAGTT	GTGTAGTG	3
GGGTATGG	GTGTATGG	2
TGGTAGTC	TGGTAGTG	2
GTGTACGG	GTGTACGC	2
TGGTAGGA	GGGTAGGA	2
GGGTATAC	GTGTATAC	2
GGGTAAGT	GGGTATGT	2
TGGTAGTC	GGGTAGTC	2
GTGTAAGA	GTGTAAGG	2
GGGTATGA	GGGTAGGA	2
GGGTATAC	GGCTAGGC	2
GAGTAGGA	GAGTAGGG	2
TTGTATGC	GTGTATGT	2
TTGTAGGC	CTGTAGGG	2

### Selecting candidate spacers

**Figure 8:**
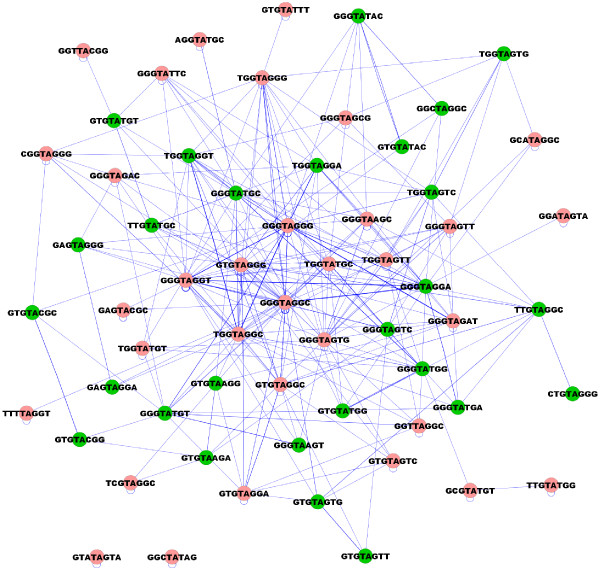
**Spacer interaction network**. Spacer interaction network highlighting promiscuity within the set of self-recombining spacers (red) and the set of non-self recombining spacers (green).

Traditionally candidate *loxP *spacer sequences with the greatest potential utility for genetic engineering will self-recombine and exhibit limited promiscuity. Some promiscuity is tolerable if the sites prone to interaction are used in constructs in a mutually exclusive manner. We visualized self and non-self spacer interactions as a network using Cytoscape [25] in order to identify spacer cross-talk and the periphery of promiscuity (Figure 8). The hubs in the middle of this interaction map are likely too promiscuous to be useful. Based on the interaction map, we identified the following peripheral 11 self and 1 non-self non-promiscuous spacers that had no more than two partners in our library [spacer sequence (# of other self-recombining partners)]: GTATAGTA (0), GGCTATAG (0), TCGTAGGC (2), GCGTATGT (2), TTGTATGG (1), GGATAGTA (1), GTGTATTT (1), AGGTATGC (1), GGTTACGG (1), TTTTAGGT (1), and GAGTACGC (1) and [GTGTACGC (2) and GTGTACGG (2)] (non-self set).

## Discussion

The present study approaches *loxP *spacer mutant screening in an unbiased manner, assuming nothing about candidate *loxP *sites save for the conserved 4 and 5 spacer positions. Candidate sites were free to recombine with any other sequence in the pool of all possible sites. The present strategy also tested the general promiscuity of *loxP *spacer sequences. Cre-*loxP *spacer crosstalk was directly assayed since any two spacer sequences could interact in the Cre reaction. This revealed the promiscuity profile of each spacer sequence and identified the spacer sequences that were favoured by the Cre recombinase protein. By applying profiling algorithms typically used to analyze transcription factor binding sites [24], we have generated the sequence profile of the consensus, that is, the spacer most favoured by Cre recombinase (Figure 5). The most notable feature of this "sequence logo" is the abundance of guanine residues. The reason for the strong bias towards guanine content remains unclear.

A number of assumptions about Cre-*loxP *recombination were directly challenged by the present study, the first being that Cre-*loxP *recombination between identical spacer sequences is favoured over recombination between non-identical spacer sequences [6,7]. Cre-*loxP *site promiscuity is not a new observation; for example initial studies identified another *loxP *recombination site in the *E. coli *chromosome, *loxB *[22]. However, since most applications of Cre-*loxP *focus on pre-defined spacers there has been limited attention paid to promiscuity. Our data showed that for each spacer, self-recombination was not significantly greater than recombination with other spacers. Second, it has been assumed that the wild-type *loxP *spacer sequence is a favoured substrate for Cre recombinase. Although this assumption has never been explicitly stated, the majority of screens to identify novel spacers focus on point mutations to the wild-type spacer sequence. In the present study the wild-type spacer was detected in only 1 recombination reaction. This was a non-self recombination and not once did this sequence undergo self recombination (see Additional file 4). Our data show that GGGTAGGN is the sequence most favoured by Cre recombinase (Figure 5). Third, Lee and Saito argued that domains exist in the spacer region which cannot tolerate mutations due the requirements of strand exchange and resolution of the Holiday junction in the *loxP *intermediate [7]. Lee and Saito described three spacer domains, two in which homology was required for exclusive self-recombination. We did not observe this in our data as all possible positions in the spacer sequence could tolerate mismatches and proceed to recombine in our *in vitro *assay. The observation of 80 reactions in our data that had all six possible degenerate spacers mismatched suggests there are few absolute spacer homology requirements required for Cre-mediated recombination (at least in an *in vitro *setting). Finally, it has been assumed that directed, non-promiscuous recombination is best mediated by identical spacer mutants. In fact, none of our self-recombining spacer mutants that reacted more than once were non-promiscuous. Further, we identified 16 pairs of non-self recombining spacers; some of which recombined at levels similar to the top self-recombiners (Table 5). For example, the non-self recombining spacers TGGTAGGC and TGGTAGGT reacted together four times which is identical to the number of GGGTAGGG self-recombinations (Table 5, Figure 7). All spacers, save for GGGTAGGG, reacted with equal probability with a suite of spacer sequences however in most cases there was one spacer that had a higher number of reactions. These results question the necessity, or preference, of the Cre recombinase to react with homologous identical sequences. In future Cre-*loxP *applications it may prove reasonable to select any two sites that show specificity for each other, regardless of whether or not they share identical spacer sequence.

Several published spacer mutants have been described in the literature as self-recombining and are non-promiscuous (at least with the wild-type *loxP *spacer sequence). Some sites are extremely non-reactive with other *loxP *sites, namely *lox*2272 [7], *m3*, and *m7 *[8]. We screened our data set for published sequences (Table 1) to see if these trends were recapitulated. The wild-type spacer was detected in one reaction but no other published spacers were detected at all. We did detect a number of the spacers in the Lee and Saito data [7] but only one of their spacers, AGGTATGC or *lox*23, was detected in our self-recombining data. These results do not challenge the utility of published spacer mutants but indicate that published spacer sequences are potentially not kinetically favoured by Cre recombinase relative to other spacers. Additionally, there has been speculation in the literature that spacers at positions 2 and 7 are essential for exclusive recombination, at least between *loxP *mutants and the wild-type spacer [7,8]. In our set of self-recombining sites we detected recombination at both positions 2 and 7 indicating that mismatches at these positions resulted in spacer promiscuity (see Additional file 4). In fact, all position, but not all nucleotide, mismatch combinations were detected in the complete dataset. However, our results cannot rule out the possibility that certain combinations of specific nucleotide mismatches can block promiscuous recombination of self-recombining spacers.

When using inverted repeat mutants the recombination reaction yields two different tandem *loxP *sites; one site with double (LE and RE) inverted repeat mutants and the other with wild-type sequence. These products are assumed to be resistant to subsequent Cre-mediated recombination. We screened all of our sequences for evidence of failure of the inverted repeat mutants to block further rounds of recombination. We found only three examples of additional recombination within our set of 3,124 successful reactions. The fact that even the most prolific spacer sequences such as GGGTAGGC did not react with the double mutants suggested that products were stable post-recombination.

There is a need for functional genomics tools that can expand the number of transgenic DNA segments that can be specifically incorporated into a target sequence. For example, synthetic biology applications require a mechanism to stitch together disparate DNA molecules from various sources in an ordered, controlled manner. Serializing RMCE or insertional recombination via inverted repeats has been limited by the small number of stable, non-promiscuous *loxP *sites identified to date. Furthermore, previous attempts to discover novel *loxP *spacers have focused on mutants slightly different than, and non-reactive with, the wild-type sequence. In this work we deploy a high-throughput pipeline that rapidly identifies novel *loxP *spacers, regardless of sequence composition, with stability post-recombination and limited promiscuity. The set of 12 spacer sequences detected with these properties have an increased probability of successfully tolerating multiple *loxP *recombinations *in vivo *and, therefore, these sites have potential utility.

## Conclusion

In this study we introduce a novel, high-throughput assay to rapidly screen for successful recombination between *loxP *spacers using standard molecular biology techniques and high throughput sequencing. Using the results from 3,124 successful reactions, we provide the first profile and comprehensive analysis of sequence spacers favouring Cre-*loxP *recombination. Increasing the guanine content at all spacer positions save for position 8 resulted in increased recombination reactions. Interestingly, self-recombination is not preferred over non-self recombination and the collection of known *loxP *spacer mutants do not appear to be favoured by Cre recombinase. We have identified novel self and non-self recombining *loxP *spacer sites that expand the repertoire of sites available for further study and potential *in vivo *recombination applications. Further, given that these sites have been shown to be functional in combination with inverted repeat mutations, they should be useful for mediating direct serial insertions into a given target construct, chromosome or genome.

## Methods

### Generating two pools of LE/RE-spacer mutants

Two oligonucleotides were designed that contained *loxP *sites with six degenerate spacer nucleotides (positions 1,2,3,6,7,8) and two central fixed spacer nucleotides (4,5) (Table 2). One of the two oligonucleotide pools (LE) contained the *lox*71 left arm mutant sequence and the other (RE) contained the *lox*66 right arm mutant sequence [12]. These oligonucleotides were synthesized (Invitrogen), purified by polyacrylamide gel electorophoresis, and double-stranded product was generated by PCR.

### Generating a library of circularized LE-spacer mutants

The amplified, double stranded LE-spacer fragment was double digested with EcoRI and HindIII, agarose gel purified, and ligated to EcoRI/HindIII linearized and agarose gel purified pUC19 vector to create a library of LE mutant sequences (Figure 1). Ligation products were transformed into DH10B cells (Invitrogen) and amplified on 2xYT solid media. Supercoiled plasmid DNA was isolated by alkaline lysis and then subsequently agarose gel purified.

### The recombination reaction

A 20 μL *in vitro *recombination reaction was set up with approximately 300 ng of supercoiled LE/pUC19 plasmid, 30 ng of double stranded RE PCR product, 200 ng of CRE Recombinase (BD Bioscience) and 1x (final concentration) Cre Recombinase Buffer (BD Biosciences). The reaction was incubated overnight at room temperature and the desired 1.8 kbp recombination products were agarose gel purified and amplified by PCR using the RE_NotI_Forward and RE_NotI_Reverse primers (Table 2).

### Sequencing recombination reaction products

The amplified recombination products were digested with NotI, agarose gel purified and re-circularized with T4 DNA ligase to generate a library of paired *loxP *recombination products in pUC19 vector. A total of 5,670 clones were sequenced with M13 Reverse sequencing primer using previously described methods [21]. Vector sequences were removed and reads were quality trimmed to the Q20 standard [26].

### Identifying successful recombinants

Sequence data was analyzed using Perl v5.6.1 and MySQL v10.8 Distribution 3.23.21-beta (for pc-linux-gnu). A Perl script termed pair_count4.pl (See Additional file 3) identified recombinants within sequence traces that contained two *loxP *sites and applied the recombination model (Figure 3) to infer the sequence of the input oligonucleotides. Successful recombinants contained serial *loxP *sites with one containing the *lox*66 and *lox*71 inverted repeat mutants and another with the wild-type inverted repeats. The inverted repeats flanked an 8 bp spacer region. All analysis details and scripts are provided online (see Additional file 5).

### Properties of recombinant spacers

Spacer sequences from the sequencing reads and the inferred input sequences were stored in a MySQL database (see Additional file 5). From these tables, all unique input spacers were identified. For each unique spacer, the number of self-recombining reactions, non-self recombining reactions, and total interactions were stored in MySQL tables. All successful recombinant spacer sequences were used to build a sequence logo representing the preferred nucleotide for Cre recombinase at each position in the spacer. A local Java implementation of the "sequence logo" algorithm [24] was used to generate a graphic representing the Cre recombinase binding site profile. Information content of each base is defined as I = sum × frequency (nt) × log(2) (4 × frequency (nt)). Therefore the height (H) of each base in the logo is: H = frequency (nt) × I. The effect of varying bases at each particular position in the spacer was evaluated by a Perl script that calculated the percent of the maximum recombination achieved by each polymorphism (observed/total) (see Additional file 5). This was calculated for all single nucleotide combinations flanking the axis of symmetry (positions 4,5) that were present in our data. Cytoscape, a software package for visualizing biomolecular interaction networks [25], was used to map out the promiscuity profile of each self-and non-self recombining spacer.

## Authors' contributions

PIM conceived of the study, contributed to study design, performed bioinformatics and statistical analysis of the data and wrote the manuscript. DES contributed to study design and performed high throughput sequencing. RAH contributed to study design, performed the *in vitro *recombination and library construction work, provided direction to data analysis, and edited the manuscript. All authors approved the final manuscript.

## Supplementary Material

Additional File 1 **RH017.fasta.all.cr – Multiple FASTA file**. Multiple FASTA file containing output from the sequencing reactions generated by the M13 Reverse primer. Click here for file

Additional File 2 **RH017.fasta.all.c21 – Multiple FASTA file**. Multiple FASTA file containing output from the sequencing reactions generated by the M13 Forward primer. Click here for file

Additional File 3 **pair_count4.pl – Perl script to extract spacer sequences from sequence reads**. This file is a Perl script that detected successful recombinants from the sequencing reads. Spacer sequence was extracted and immediately deposited into a MySQL database. Click here for file

Additional File 4 **Table S1 – Table_S1_mismatch_analysis.xls – Sequence of spacers participating in recombination**. This file is an Excel spreadsheet all of the *loxP *spacers that successfully recombined. Contains pre- and post- recombination sequence as inferred from the location of the scissile bonds and reaction mechanism (see Figure 3 for details). This is the effective output of the pair_count4.pl script. All data analysis was performed on the inferred spacer sequences as they were considered the input oligonucleotides into the reaction. Click here for file

Additional File 5 **Analysis_scripts.zip – zip file containing all analysis scripts**. All SQL, Perl and shell scripts used in this analysis. Detailed explanation of their functionality given in the README.txt file. Click here for file
